# Efficacy of customised foot orthoses in the treatment of Achilles tendinopathy: study protocol for a randomised trial

**DOI:** 10.1186/1757-1146-2-27

**Published:** 2009-10-24

**Authors:** Shannon E Munteanu, Karl B Landorf, Hylton B Menz, Jill L Cook, Tania Pizzari, Lisa A Scott

**Affiliations:** 1 Musculoskeletal Research Centre, Faculty of Health Sciences, La Trobe University, Bundoora 3086, Victoria, Australia; 2 Department of Podiatry, Faculty of Health Sciences, La Trobe University, Bundoora 3086, Victoria, Australia; 3 School of Exercise and Nutrition Sciences, Faculty of Health, Medicine, Nursing and Behavioural Sciences, Deakin University, Burwood 3125, Victoria, Australia; 4 School of Physiotherapy, Faculty of Health Sciences, La Trobe University, Bundoora 3086, Victoria, Australia

## Abstract

**Background:**

Achilles tendinopathy is a common condition that can cause marked pain and disability. Numerous non-surgical treatments have been proposed for the treatment of this condition, but many of these treatments have a poor or non-existent evidence base. The exception to this is eccentric calf muscle exercises, which have become a standard non-surgical intervention for Achilles tendinopathy. Foot orthoses have also been advocated as a treatment for Achilles tendinopathy, but the long-term efficacy of foot orthoses for this condition is unknown. This manuscript describes the design of a randomised trial to evaluate the efficacy of customised foot orthoses to reduce pain and improve function in people with Achilles tendinopathy.

**Methods:**

One hundred and forty community-dwelling men and women aged 18 to 55 years with Achilles tendinopathy (who satisfy inclusion and exclusion criteria) will be recruited. Participants will be randomised, using a computer-generated random number sequence, to either a control group (sham foot orthoses made from compressible ethylene vinyl acetate foam) or an experimental group (customised foot orthoses made from semi-rigid polypropylene). Both groups will be prescribed a calf muscle eccentric exercise program, however, the primary difference between the groups will be that the experimental group receive customised foot orthoses, while the control group receive sham foot orthoses. The participants will be instructed to perform eccentric exercises 2 times per day, 7 days per week, for 12 weeks. The primary outcome measure will be the total score of the Victorian Institute of Sport Assessment - Achilles (VISA-A) questionnaire. The secondary outcome measures will be participant perception of treatment effect, comfort of the foot orthoses, use of co-interventions, frequency and severity of adverse events, level of physical activity and health-related quality of life (assessed using the Short-Form-36 questionnaire - Version two). Data will be collected at baseline, then at 1, 3, 6 and 12 months. Data will be analysed using the intention to treat principle.

**Discussion:**

This study is the first randomised trial to evaluate the long-term efficacy of customised foot orthoses for the treatment of Achilles tendinopathy. The study has been pragmatically designed to ensure that the study findings are generalisable to clinical practice.

**Trial registration:**

Australian New Zealand Clinical Trials Registry Number: ACTRN12609000829213.

## Background

Achilles tendinopathy is a common musculoskeletal disorder, accounting for between 8-15% of all injuries in recreational runners [1-3] and having a cumulative lifetime incidence of approximately 6% in non-athletes and 24% in athletes [4]. Interestingly, one-third of patients with chronic Achilles tendinopathy are not physically active [5] and Achilles tendinopathy is more common in those aged 35 years and over [6]. In some settings, approximately 30% of patients who present with this condition require surgical treatment [7]. Since physical inactivity is a risk factor for many multisystem diseases [8], Achilles tendinopathy may lead to poorer overall health and greater morbidity, not just sporting inconvenience.

Numerous non-surgical treatments have been proposed for the treatment of Achilles tendinopathy including: footwear modification, activity modification and weight reduction [9]; ultrasound and manual therapy techniques [10]; flexibility and strengthening exercises [11]; extracorporeal shock wave therapy [12]; as well as various pharmacological agents including corticosteroids, heparin, dextrose, aprotinin, glyceryl trinitrate and sclerosing agents [10]. However, many of these treatments have a poor or a non-existent evidence base [10].

Eccentric calf muscle exercise is an emerging treatment intervention for the management of tendinopathy, particularly for the Achilles tendon. Although the mechanism of action [13] and optimum dosage (speed of contractions, duration and frequency) for rehabilitation using eccentric calf muscle exercises has yet to be clearly established, up to three sets of fifteen repetitions, performed twice daily for at least eleven weeks of a twelve week period has been shown to be effective in high quality studies [14]. Recent systematic reviews have concluded that eccentric calf muscle exercise is a promising intervention and has the most evidence to reduce pain in those with chronic Achilles tendinopathy [15,16]. In a review of 9 clinical trials, eccentric calf muscle exercise reduced pain by an average of 60% [15]. However, eccentric calf muscle exercise alone may not be effective in all people, as up to 40% of those with Achilles tendinopathy do not improve with this intervention [12], and eccentric calf muscle exercise has been shown to be less effective in inactive people [17]. Also, major criticisms of current research in this area are the lack of use of disease-specific functional outcome measures and inadequately powered study designs [15,18]. Nevertheless, eccentric calf muscle exercise is currently considered the best evidence-based intervention available.

A further intervention that has been advocated for the treatment of Achilles tendinopathy is foot orthoses [11,19-21]. The classical theoretical mechanism for the use of foot orthoses for this condition is that they align the calcaneus to a more vertical position and reduce bending stress applied to the Achilles tendon, particularly in a pronated foot [22]. However, this theory has recently been challenged by recent findings that a more laterally directed force distribution during early stance followed by a more medially directed force distribution during late stance may be a risk factor for Achilles tendinopathy [2]. Further, recent studies indicate that the mechanical effects of foot orthoses are non-specific and small, and that their mechanism of action is likely to be more complicated, possibly involving neuromotor effects [23-26]. Therefore, there is currently a lack of evidence to explain the mechanism by which foot orthoses exert their effects when used to treat Achilles tendinopathy.

Despite the mechanism by which foot orthoses exert their effects being unclear, there is evidence from a small number of studies to suggest that they may reduce symptoms in those with Achilles tendinopathy [21,27,28]. Mayer and co-workers [27] performed a randomised clinical trial comparing the effects of four weeks of physiotherapy treatment (n = 11) (total of 10 treatments: deep friction massage, ultrasound, ice and sensory motor training consisting of balance and eccentric exercises) versus semi-rigid customised foot orthoses (n = 10) versus a no-intervention control group (n = 10) in athletes with Achilles tendinopathy. Outcome measures for symptoms were the 'Pain Disability Index' (PDI) and 'Pain Experience Scale' (PES) scores. After four weeks, there were significant differences between the groups. Both the physiotherapy and customised foot orthoses groups, compared to the control group, demonstrated significantly greater improvements in pain, as measured by the PDI and PES scores.

In a retrospective case-series study, Donoghue and colleagues [21] evaluated the effectiveness of customised high-density ethylene vinyl acetate (EVA) foot orthoses to alter lower limb kinematics and reduce pain in athletes with chronic Achilles tendinopathy who displayed a pronated foot type (n = 12). Participants reported a mean improvement of 92 ± 16% in symptoms with the use of the orthoses.

Whilst these studies suggest that customised foot orthoses can reduce symptoms in those with Achilles tendinopathy, they both have a number of limitations. First, the sample sizes used were small. Second, the study by Donoghue and colleagues [21] was retrospective and lacked a control group for comparison. Third, neither study used blinding of the participants or assessors which could have lead to bias. It is therefore possible that the positive symptom-modifying effects of the foot orthoses measured in these studies may have been overestimated. Also, another criticism of current research in this area is the lack of use of disease-specific functional outcome measures [15,17]. Finally, in the study by Mayer and co-workers [27], the physiotherapy and custom foot orthoses interventions were used mutually exclusive of one another. In clinical practice, the two interventions are likely to be used concomitantly.

In light of the limitations of previous studies, the aim of this project is to conduct a participant-blinded randomised trial to determine the effectiveness of customised foot orthoses on (i) pain, function and activity (using the Victorian Institute of Sport Assessment - Achilles questionnaire) [29]; (ii) participant perception of change in symptoms; (iii) comfort of the foot orthoses; (iv) use of co-interventions; (v) frequency and severity of adverse events; (vi) level of physical activity in previous week; and (vii) health-related quality of life (using the Short-Form-36 questionnaire) in people with Achilles tendinopathy. The study protocol is presented in this paper, consistent with the recommendations of Editorial Board of BioMed Central [30].

## Methods

### Design

**Figure 1:**
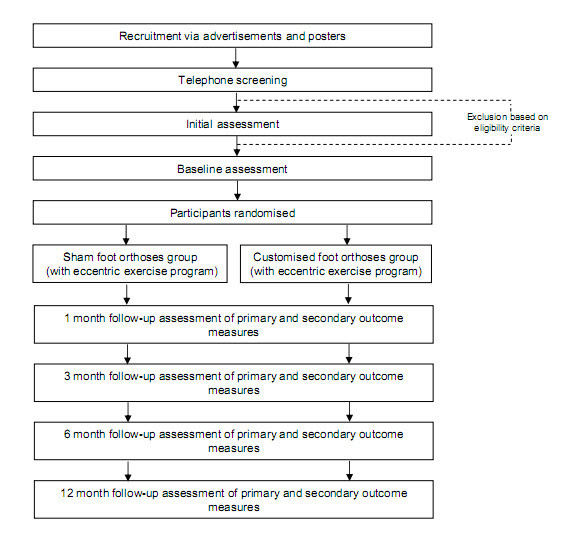
**Design of study**.

This study is a parallel group, participant blinded, randomised controlled trial with a 12 month follow-up (Figure 1). Participants will be randomised to a control group (sham foot orthoses) or an experimental group (customised foot orthoses). To ensure all participants, who will have some level of pain and disability, receive some form of intervention, both groups will be prescribed the same eccentric calf muscle exercise program. This design covers any ethical concerns of not treating participants in pain, but will allow the effectiveness of customised foot orthoses to be evaluated. Allocation to either of the intervention groups will be achieved using a computer-generated random number sequence. The allocation sequence will be generated and held by an external person not directly involved in the trial. Concealment of the allocation sequence will be ensured as each participant's allocation will be contained in a sealed opaque envelope. Envelopes will be made opaque by using a sheet of aluminium foil inside the envelope. In addition, a system using carbon paper will be employed so the details (name of participant and date of recruitment) are transferred from the outside of the envelope to the paper inside the envelope containing the allocation prior to opening the seal.

### Participants

The Human Studies Ethics Committee at La Trobe University (Human Ethics Committee Application No. 08-114) has approved the study. Written informed consent will be obtained from all participants prior to their participation.

People with Achilles tendinopathy will be recruited from a number of sources:

(i) Advertisements in relevant Melbourne (Australia) newspapers;

(ii) Mail-out advertisements to appropriate health professionals in Melbourne;

(iii) Advertisements using relevant internet web-sites;

(iv) Posters displayed in local community centres, sporting clubs and universities located in Melbourne.

Respondents will initially be screened by telephone interview to ensure they are suitable for the study. Suitable individuals will then be invited to participate in the study and attend an initial assessment.

To be included in the study, participants must meet the following inclusion criteria:

(i) Aged 18 to 55 years;

(ii) Have symptoms in the Achilles tendon of one lower limb only for at least 3 months duration;

(iii) Be literate in English and able to complete the Victorian Institute of Sport Assessment - Achilles (VISA-A) questionnaire [29];

(iv) Score less than 80 on the VISA-A questionnaire [29];

(v) Regularly use footwear that can accommodate customised foot orthoses. This is defined as using footwear that can accommodate foot orthoses for at least 90% of the time during weightbearing activities [31];

(vi) Be willing to not receive any physical therapy on the involved Achilles tendon(s) or trial of foot orthoses or bracing (other than those allocated in the current study) during the study period.

Achilles tendinopathy will be diagnosed from a clinical assessment as well as from a musculoskeletal ultrasound assessment using the following criteria [32-34]:

(i) Insidious onset of pain in the Achilles tendon region that is aggravated by weightbearing activities and worse in the morning, and/or during the initial stages of weightbearing activities;

(ii) Pain and swelling located 2-6 cm proximal to the Achilles tendon insertion (as described by patient and palpated by the investigator);

(iii) Musculoskeletal ultrasound imaging of the Achilles tendon showing local thickening (anterior-posterior) and/or irregular fibre orientation and/or irregular tendon structure with hypoechoic areas and/or vascularisation within the mid-portion of the Achilles tendon.

Exclusion criteria for participants in this study will be [12,17]:

(i) Previous Achilles tendon surgery in the symptomatic lower limb;

(ii) Previous Achilles tendon rupture in the symptomatic lower limb;

(iii) Previous lower limb trauma that has caused structural imbalance (e.g. ankle fracture);

(iv) Osseous abnormality of the ankle (e.g. anterior or posterior tibio-talar osteophytes);

(v) Inflammatory arthritis (e.g. ankylosing spondylitis);

(vi) Metabolic or endocrine disorders (e.g. type I or II diabetes);

(vii) Neurological disorders (e.g. Charcot-Marie-Tooth disease);

(viii) Previous breast cancer and/or use of oestrogen inhibitors;

(ix) Treatment with foot orthoses, heel lifts or eccentric calf muscle exercises within the previous 3 months;

(x) Disorders of the Achilles tendon that are not mid-portion tendinopathy (such as paratendinitis and insertional Achilles tendon disorders);

(xi) Taken fluoroquinolones within the previous 2 years;

(xii) Injection of local anaesthetic, cortisone or other pharmaceutical agents into the Achilles tendon or surrounding area within the previous 3 months;

(xiii) Injury or pathology of the foot, knee, hip and/or back or any condition that, in the opinion of the investigators, may interfere with participation in the study.

Investigators will enquire about the above inclusion/exclusion criteria during the initial telephone contact with the potential participant and at the initial appointment.

### Intervention

Participants will be randomised to one of two groups: an intervention group (customised foot orthoses) or a control group (sham foot orthoses). Both groups will receive eccentric calf muscle exercises. To maintain blinding of participants, they will be advised that they will receive one of two types of 'shoe inserts' during the study.

Data collection and the interventions will be administered by 2 experienced qualified podiatrists. These podiatrists will have attended two seminars for explanation and discussion of the intervention protocols prior to the study recruitment. During the seminars, the podiatrists will receive further training regarding the administration of the eccentric exercise program by qualified sports physiotherapists (JLC and TP) who have extensive experience in the management of Achilles tendinopathy. A detailed manual outlining study procedures will be provided to all project investigators.

An appointment will be given to all participants one month after receiving their intervention to review the participant's condition, assess compliance with the intervention, ensure the foot orthoses are comfortable and confirm that proper form and technique of the eccentric calf muscle exercises is being adhered to.

Participants will be requested to refrain from other forms of physical therapy intervention, not use any mechanical interventions (apart from the foot orthoses provided as part of this study), and not to consume non-steroidal anti-inflammatory medications. They will be allowed to take 500 mg of paracetamol on an ad-hoc basis if the tendon is painful.

The advice given to the participants with regard to the amount of activity allowed during the study will be based on the pain-monitoring model [35]. This approach allows participants with Achilles tendinopathy to continue with some level of activity during rehabilitation and shows equivalent outcomes to programs that involve complete rest from the aggravating activity with no negative effects [35]. Using this approach, participants will be advised that they can continue their activities after receiving their intervention. However, Achilles tendon pain should not be allowed to reach level 5 on the visual analogue scale (VAS), where 0 is no pain and 10 is worst pain imaginable, during the activity. The pain after the activity can reach 5 on a VAS but should have subsided by the following morning. Pain and stiffness in the Achilles tendon should not increase from week to week [35].

### Customised foot orthoses

**Figure 2:**
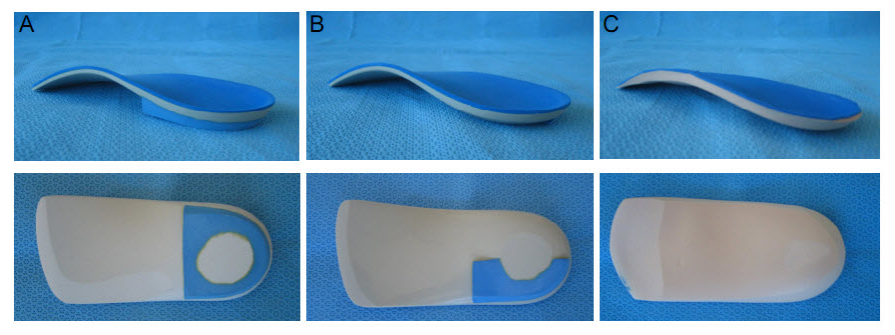
**The customised foot orthoses for a foot with a normal foot posture (FPI score of +1 to +7) (A), a supinated foot posture (FPI score of 0 or less) (B) and sham foot orthoses (C) used in this study**. Upper panels show posterior-medial view and lower panels show plantar view.

Participants randomised to this intervention group will receive customised foot orthoses for both feet. The basic contour of the shell of all of the customised foot orthoses will be based on the description of the modified Root style of orthoses [36], and posted to vertical [36]. This style of foot orthoses has been shown to be most commonly prescribed by Australian and New Zealand podiatrists [37,38]. All customised foot orthoses will be manufactured from polypropylene with a 400 kg/m^3 ^ethylene vinyl acetate (EVA) rearfoot post and a shell-length covering fabric (Nora^® ^Lunasoft SL 2 mm) (Figure 2a).

The foot orthoses will be further customised using the information obtained from assessment of the foot posture of each foot and the participants' body mass. The foot posture will be measured using the Foot Posture Index-6 (FPI), which is a valid and reliable tool [39]. The FPI consists of six specific criteria: talar head palpation, supralateral and infralateral malleolar curvature, calcaneal frontal plane position, prominence in the region of the talonavicular joint, medial arch height and abduction and adduction of the forefoot on the rearfoot. Each FPI criterion is scored on a 5-point scale (range, -2 to +2). The six scores obtained are then summated to give an overall score of foot posture. The summated score has the potential to range from -12 (highly supinated) to +12 (highly pronated) [39]. Feet that are assessed to have an FPI of (i) 0 or less will be considered to be supinated, (ii) +1 to +7 will be considered normal, and (iii) +8 or greater will be considered to be pronated [40].

Those feet that are assessed to be pronated, defined as obtaining an FPI summated score of +8 or greater [40], will have a 4.0 mm Kirby medial heel skive (15 degree varus heel wedge) incorporated into their orthosis [41]. This modification is thought to increase the anti-pronation effect of the foot orthosis. The thickness of the polypropylene used for the customised foot orthoses will vary depending on the body mass of the participant. For those feet assessed as being normal or pronated, the thickness of the polypropylene will be 4.0 mm for participants with a body mass of less than 75 kg, and 4.5 mm for participants with a body mass equal to or greater than 75 kg [42].

Those feet that are assessed to be supinated, defined as obtaining an FPI summated score of 0 or less [40], will receive an 'anti-supination' foot orthosis, based on the description by Burns et al. [43] and Hertel et al. [44], but with some modifications. This foot orthosis will have the medial half of the rearfoot post removed [43,44] and be fabricated from a relatively more flexible polypropylene (Figure 2b) [43]. However, the thickness of the polypropylene will be determined by the participant's body mass. The thickness of the polypropylene will be 3.0 mm for participants with a body mass of less than 75 kg, and 4.0 mm for participants with a body mass equal to or greater than 75 kg. The Burns et al. [43] original description of this device also used a padded full-length top cover as it was designed to reduce excessive plantar pressures. In our study, the anti-supination foot orthosis will not have a padded top cover as the aim of our orthotic intervention is to provide a pronatory force to the foot (resist supination), rather than reduce plantar pressures.

The customised foot orthoses will be manufactured and donated by a commercial laboratory (Footwork Podiatric Laboratory Pty Ltd, Victoria, Australia). Once fabricated, the customised foot orthoses will be dispensed to participants two weeks after the initial appointment. Participants will be advised to remove any existing inner soles from their shoes. The participant will also be given a handout that provides instructions for using the orthoses, including adjustment to them.

### Sham foot orthoses

This condition will act as a control for the customised foot orthoses intervention and be provided for both feet of each participant. The sham foot orthoses will be made from 4.0 mm thick ethylene vinyl acetate (EVA) with a density of 90 kg/m^3 ^and have an identical covering fabric. The shape of the sham foot orthoses will be derived from being vacuum moulded over a standard cast which has been obtained from replication of a prefabricated foot orthosis (Prothotic S, Footech Orthotics™, The Orthotic Laboratory Pty Ltd, Victoria, Australia). The sham foot orthoses will have similar shape to the customised foot orthoses (Figure 2c), however, they will not provide any mechanical support as the arch will flatten upon minimal compressive force. This form of device has been used previously as a sham condition in a previous trial [38]. The sham foot orthoses will be dispensed to the participant two weeks after the initial appointment. The participant will also be given the same handout that provides instructions for using the sham foot orthoses as participants that receive the customised foot orthoses.

### Eccentric calf muscle exercise program

The eccentric calf muscle exercise program will be performed by all participants and is based on the method by Alfredson et al. [45]. Participants will be given an information package that includes a booklet and DVD with instructions on performing the eccentric exercises for Achilles tendinopathy [see Additional files 1, 2, 3, 4]. The program is described below:

The participants will be instructed to do eccentric calf muscle exercises 2 times daily, 7 days per week, for 12 weeks. Two types of eccentric calf muscle exercises will be used. The calf muscle will be eccentrically loaded both with the knee straight to maximise the activation of the gastrocnemius muscle, and also with the knee bent to maximise the activation of the soleus muscle. Each of the two exercises will include 15 repetitions done in 3 sets (i.e. 3 sets of 15 repetitions). The participants will be told that muscle and tendon soreness during the first 4 weeks of training is to be expected. After 12 weeks, the participants will be required to perform the exercises once daily, 3 days per week for the remainder of the study (12 months).

In the beginning, the loading will consist of bodyweight and the participants will be standing with all their bodyweight on the injured leg. Participants will stand with their heels over the edge of a step. From an upright body position and standing with all bodyweight on the forefoot and the ankle joint in plantar flexion, the calf muscle will be loaded by having the participant lower the heel beneath the forefoot. Only eccentric loading the calf muscle will be allowed: minimal concentric loading will be performed. Instead, the non-injured leg will be used to return to the start position. If participants are unable to load their injured leg with all of their bodyweight, they will be advised to use their non-injured leg to assist until they are able to load their injured leg with all of their bodyweight. Participants will be advised to perform the exercise even if they experience pain. However, they will be warned to stop the exercise if the pain becomes disabling. When the exercise can be completed with no pain or discomfort, participants will progress to performing the exercise with a weighted back-pack containing 5 kg of mass (bricks, books etc). They will be advised to continue to add mass in multiples of 5 kg, up to a maximum of 20 kg, if they do not experience pain in the Achilles tendon by the end of the third set of the eccentric calf muscle exercises. Participants will be advised to apply ice on the affected area of the Achilles tendon for 15 minutes after completion of an exercise session.

### Assessments

#### Initial assessments

An initial assessment will be performed to determine the eligibility of participants for this study. Participants will complete a questionnaire to obtain data concerning the presentation of symptoms (lower limb affected, location, characteristics and duration of symptoms). Demographic and anthropometric data will also be collected including the age, gender, waist and hip circumference [46], height and mass of participants. Data concerning the participants' sporting activities (including type, frequency and duration) will also be obtained.

Foot posture will be determined for both feet of each participant during the initial assessment. The foot posture will be measured using the Foot Posture Index-6 (FPI), which has been previously described [39].

A pair of neutral suspension plaster casts of both feet with participants positioned non-weightbearing (prone) will be taken to allow fabrication of the customised foot orthoses. Plaster casts will be taken as previously described [47]. To maintain blinding of participants, all participants will have plaster casts taken of their feet.

To confirm that participants have Achilles tendinopathy, an ultrasound assessment will also be performed, as described by Leung and Griffith [34]. A qualified sonographer, who will be aware of the clinical status of the participants, will perform the examinations using grey scale settings of an ultrasound machine with a 13.5 MHz linear transducer (Siemens Anatares, Siemens, Germany). The participant will be positioned prone with the feet hanging free in a neutral position over the end of the examination table. Tendon and paratendinous structures will be imaged in both transverse and longitudinal planes [34]. Assessments of tendon dimensions, echogenicity, echotexture, and presence of calcifications will be performed. Paratendinous structures (subcutaneous tissue, paratenon, Kager's fat pad, retrocalcaneal and Achilles/precalcaneal bursae, calcaneal cortical outline) will also be assessed. The dimensions of the Achilles tendon (both maximum antero-posterior diameter and cross-sectional area) will be measured at 3 sites: the musculotendinous junction, just proximal to the calcaneal insertion, and at the midpoint between the previous two sites [34]. Transverse sections will be used to measure tendon thickness (with the electronic calipers) and cross-sectional area (by tracing of the tendon's outline) [33,34]. After assessment of grey-scale characteristics, colour Doppler assessment of the entire tendon will be performed in both transverse and longitudinal planes (to assess for neovascularisation). All images will be recorded for subsequent review by one of the study investigators.

Participants who have local thickening [33] and/or irregular fibre orientation and/or irregular tendon structure with hypoechoic areas and/or neovascularisation within the mid-portion of the Achilles tendon (1 or more vessels visible within the Achilles tendon) [48]) will be deemed to have Achilles tendinopathy [12]. Participants will not be excluded if they have any of the aforementioned sonographic features accompanied by fluid in the retrocalcaneal bursae (up to 4.0 mm), focal calcifications, paratenon thickening (considered to be present if the paratenon measures more than 2.0 mm in thickness [34]), or calcaneal cortical anomalies (such as spurring). These features have been shown to concomitantly exist in those with Achilles tendinopathy, and may also exist in asymptomatic people [34].

### Baseline assessments and outcome measures

Participants who are eligible for the study will be invited to attend a baseline assessment. During the baseline assessment, participants will undergo primary and secondary outcome measurements prior to receiving their intervention. Outcome measurements (primary and secondary) will occur at five time-points at baseline, 1, 3, 6 and 12 months. The outcome measurements at 6 and 12 months will occur via questionnaires mailed to participants at these times. A pre-paid envelope will be included to facilitate the return of these questionnaires. Participants will be free to contact the researchers at any time during the study. The researchers involved in the data entry phases of this study will be blinded as to the intervention the participants have been allocated to.

Participant compliance with the eccentric calf muscle exercise will be measured by daily registration in the form of a diary which will be returned to the investigators at 1 and 3 months. Participants will be required to document the number of repetitions, sets and load performed for each day of the exercise program (12 weeks). Compliance at 3 months will be determined by the number of exercise sessions performed per week (e.g., 100% compliance = 14 sessions per week) [49]. Compliance will be classified into four categories. When <25% of the exercises are performed, participant compliance will be classified as poor, between 25 and 50% it will be moderate, between 50 and 75% will be classified as good and >75% will be classified as excellent. The number of participants classified as demonstrating 'poor or moderate', 'good', and 'excellent' compliance will be documented for each intervention group [50]. The compliance with the customised foot orthoses or sham orthoses will be assessed at 1, 3, 6 and 12 months. Participants will provide information concerning the number of hours per day and number of days they have worn their foot orthoses during the past week. The use of the foot orthoses for sports and exercise will also be determined using a 5-point Likert scale. The scale will ask "How much of the time have you worn the shoe inserts during your sport or other physical activity in the previous week?", and have the following five responses: "all of the time", "most of the time", "some of the time", "a little of the time" and "none of the time". For the purpose of analysis, this scale will then be dichotomised according to compliance for exercise, where 'compliance for exercise' is defined as most or all of the time on this scale.

### Primary outcome measures

The primary outcome measure will be the total score of the Victorian Institute of Sport Assessment - Achilles (VISA-A) questionnaire. The VISA-A questionnaire has been developed primarily to assess the clinical severity of Achilles tendinopathy [29]. The VISA-A questionnaire evaluates three domains that are clinically relevant to patients: pain, function and activity. The VISA-A questionnaire has been validated (construct validity), and shows good test-retest reliability [29]. Other strengths of the VISA-A questionnaire are that it can be self-administered, is likely to be sensitive to small changes occurring over a medium duration of time and has previously been used to monitor the clinical severity of Achilles tendinopathy in response to treatments [12,17,29,35].

The VISA-A questionnaire contains 8 questions that cover 3 domains of pain (questions 1 to 3), function (questions 4 to 6), and activity (questions 7 and 8). Questions 1 to 7 are scored out of 10, and question 8 has a maximum score of 30. Scores are summated to give a total score out of 100. Higher scores indicate less severe Achilles tendinopathy. Therefore, an asymptomatic person would score 100 [29].

### Secondary outcome measures

The secondary outcome measures will be:

#### (i) Participant perception of treatment effect

The perception of treatment effect will be assessed using a 5-point Likert scale. The scale will ask "How has the pain in your Achilles tendon(s) changed since you received treatment?", and have the following five responses: "marked worsening", "moderate worsening", "same", "moderate improvement", and "marked improvement". For the purpose of analysis, this scale will then be dichotomised according to success, where 'success' is defined as marked or moderate improvement on this scale [51,52].

#### (ii) Comfort of the interventions (customised foot orthoses and sham foot orthoses)

The comfort of the customised foot orthoses and sham foot orthoses will be assessed using a 150 mm visual analogue scale with the left end of the scale (0 mm) labelled "not comfortable at all" and the right end of the scale (150 mm) labelled "most comfortable imaginable". Participants will be asked "Please indicate the comfort of your shoe inserts compared to when they are not in your shoes, the further the right the more comfortable the shoe inserts". The reliability of this scale has been shown to be good (ICC = 0.799) when a protocol including a control condition is used [53].

#### (iii) Use of co-interventions to relieve pain at the Achilles tendon(s)

The number of participants who consume rescue medication (i.e., paracetamol) and mean consumption of rescue medication to relieve pain at the Achilles tendon(s) (mean grams of paracetamol/participant/month] will be assessed using a medications diary that participants will self-complete [54-56]. The diary will be returned to the investigators at monthly intervals for analysis.

A questionnaire regarding the use of other treatments to relieve pain at the Achilles tendon(s) by participants will be completed at 1, 3, 6 and 12 months. Other treatments will include oral non-steroidal anti-inflammatory medication, visits to health-care practitioners (general practitioners, specialists and allied health professionals such as physiotherapists and podiatrists), changes to foot orthoses or wedging, massage, acupuncture, complementary medicine (such as osteopaths and naturopaths), topical medicaments (such as rubefacients or topical non-steroidal anti-inflammatory medication), taping or bracing [57]. Participants will also be questioned to determine if they have changed their footwear they normally wear (worn for everyday or sporting activities) to accommodate their foot orthoses.

#### (iv) Frequency and severity of adverse events

The frequency (number of participants affected and number of cases), types (including rubbing or blistering of the feet or ankles, pain in the feet, lower limbs or other part(s) of the body) and severity (mild, moderate or severe as rated by the participant) of adverse events in each intervention group during the trial will be recorded using a questionnaire that participants will complete at 1, 3, 6 and 12 months. An open-response type format will also be available for participant responses.

#### (v) Level of physical activity in the previous week

The level of physical activity in the previous week will be evaluated with a questionnaire, the 7-day Recall Physical Activity Questionnaire [58]. This questionnaire records all physical activities (work as well as leisure and household activities) during the preceding week. The questionnaire involves quantifying the time (hours) spent in moderate, hard and very hard activities during the preceding 7 days. The time (hours) spent in each activity is then multiplied by its metabolic equivalent (MET) where 1 MET is the energy expended by a person while sitting at rest (equal to 1 kilocalorie per kilogram per hour). The total calories (kilocalories) of energy expended per kilogram of body weight can then be calculated. Kilocalories per day (for the participant) can then be derived by multiplying the kilocalories per kilogram by the participant's body weight and dividing this by 7. This questionnaire has been shown to have good reliability and validity [58] and has been used previously in studies investigating the effects of interventions for lower limb musculoskeletal pathology [51,52].

#### (vi) Health-related quality of life

The Short-Form-36 (Version two) (SF-36) questionnaire will be used to assess health-related quality of life. The SF-36 is a 36 question survey that measures eight health concepts most affected by disease and treatment. The eight health concepts can then be used to form two summary measures: *physical health *and *mental health*. The SF-36 has been extensively validated and is one of the most widely used instruments to measure health status. The SF-36 has sound reliability and validity [59-62].

### Sample size

The sample size for the study has been pre-specified using an *a priori *power analysis using the primary outcome measure of the total score of the VISA-A questionnaire [29]. One hundred and forty participants (i.e. 70 per group) would provide power of over 80% to detect an effect size of 10-points on the VISA-A questionnaire with the significance level set at p < 0.05. An effect size of 10 points was determined to be a clinically significant difference worth detecting [17] and a standard deviation of 20 was derived from previous reports (i.e. standard deviations derived from the VISA-A questionnaire) [12,17,29,35]. This calculation includes a 10% drop-out rate [12]. Further, we have conservatively ignored the extra precision provided by covariate analysis when estimating the sample size.

### Statistical analysis

Statistical analysis will be undertaken using SPSS version 14.0 (SPSS Corp, Chicago, IL, USA) statistical software. All analyses will be conducted on an intention-to-treat principle using all randomised participants in the groups they were originally randomised to [63-65]. Missing data will be replaced with the last score carried forward; although the authors reserve the right to review this if a significantly larger number of participants drop out of one group (15% difference between groups) [66] as this technique may falsely affect the results [67]. Standard tests for normal distribution will be used and transformation carried out if required.

Demographic and anthropometric characteristics (gender, age, mass, height, body mass index, waist-to-hip circumference ratio, sporting activities, foot posture using the FPI) will be determined at the baseline visit for each treatment group. Summary statistics will be calculated for duration of symptoms, side affected (left, right), sonographic measurements (antero-posterior thickness, cross-sectional area, presence of vascularisation, presence of irregular tendon structure with hypo-echoicity) of the Achilles tendon, as well as all primary and secondary outcome measurements for each treatment group.

Analyses will be conducted on 1, 3, 6 and 12 month outcome measures. However, the primary end-point will be change in the total score of the VISA-A questionnaire at 3 months. The continuously scored outcome measures at 1, 3, 6 and 12 months will be compared using analysis of covariance with baseline scores and intervention group entered as independent variables [68,69]. The exception to this will be the comfort of the foot orthoses interventions and compliance with the foot orthoses interventions which will be analysed using independent t-tests. Nominal and ordinal scaled data will be compared using chi-square analyses (or Fisher's exact test where appropriate) and Mann-Whitney U-tests, respectively. Effect sizes will be determined using Cohen's *d *(continuous scaled data) or odds ratios (nominal and ordinal scaled data) as appropriate. Hypothesis tests will be considered significant if p < 0.05.

## Discussion

This study is a randomised controlled trial designed to investigate the efficacy of customised foot orthoses to reduce pain and improve function in people with Achilles tendinopathy. Two studies have previously investigated the efficacy of customised foot orthoses for the treatment of pain associated with Achilles tendinopathy [21,27]. However, these studies had limitations in that the sample sizes used were small, the study protocols did not blind participants and they also lacked the use of disease-specific functional outcome measures such as the VISA-A questionnaire.

The study protocol described here will overcome these limitations. It has been designed using recognised criteria for quality assessment of randomised clinical trials [70]. The primary outcome measure will be the Victorian Institute of Sport Assessment - Achilles (VISA-A) questionnaire [29]. The secondary outcome measures will be the participant perception of change in symptoms, comfort of the foot orthoses, use of co-interventions, frequency and severity of adverse events, level of physical activity in previous week, and health-related quality of life (using the SF-36 questionnaire). Previous studies investigating the usefulness of foot orthoses for lower limb musculoskeletal pathologies have shown that the short- versus long- term symptom-modifying effects of foot orthoses may differ [38,57,71]. Thus, the use of follow-up assessments at multiple time points, up to 12 months, will allow us to more comprehensively determine the effects of the customised foot orthoses.

We have chosen to evaluate the effectiveness of customised foot orthoses in participants with Achilles tendinopathy who are undergoing an eccentric calf muscle exercise program. Eccentric calf muscle exercises have become the accepted treatment for Achilles tendinopathy. As such, other interventions such as foot orthoses would be more likely to be used in conjunction with an eccentric exercise program rather than in isolation [50,72]. Hence, our study protocol using this approach is more likely to be clinically valid. Further, as all participants have some level of pain and disability, including a calf muscle eccentric exercise program in both study groups will overcome any ethical concerns of not treating participants in pain.

At present, there are no empirically-proven guidelines for the prescription of customised foot orthoses. In light of this limitation, our customised foot orthoses prescription protocol has been developed by consensus using 3 podiatrists (SEM, KBL and HBM), all with at least 10 years clinical experience. The customised foot orthoses need to reflect what is commonly prescribed in clinical practice. As such, the technique for obtaining the impressions of the participants' feet (neutral suspension casting) and the basic design of the customised foot orthoses (modified Root style made from polypropylene for the orthotic shell material and EVA for the rearfoot posting material) have been shown to be most commonly prescribed by Australian and New Zealand podiatrists [37]. Several other variations to the basic design of the orthoses shell can also be used to improve the customisation of the orthoses [37]. We have included the medial heel skive technique (15 degree varus heel wedge) as a means of further increasing the ability of the custom foot orthoses to control pronatory forces at the foot in those feet that are pronated [41]. In contrast, those feet that are assessed as being supinated will receive foot orthoses that are modified (modified rearfoot heel post, flexible shell material) to exert an anti-supinatory force to the foot [43,44]. We considered adding heel lifts to the customised foot orthoses as this is a commonly recommended intervention for reducing Achilles tendon loading in those with Achilles tendinopathy [9]. However, we did not use this intervention as biomechanical analyses have shown that heel lifts may increase Achilles tendon loading [73].

In summary, this project is the first randomised controlled trial to be conducted to evaluate the efficacy of customised foot orthoses for reducing pain and improving function and disability in people with Achilles tendinopathy undergoing an eccentric calf muscle exercise program. The study protocol, including interventions, has been pragmatically designed to ensure that the study findings are generaliseable to clinical practice. Recruitment for the study will commence in June 2009, and we expect final results to be available in late 2010.

## Competing interests

HBM, KBL and SEM are Editor-in-Chief, Deputy Editor-in-Chief and Assistant Editor, respectively, of *Journal of Foot and Ankle Research*. It is journal policy that editors are removed from the peer review and editorial decision making processes for papers they have co-authored.

## Authors' contributions

SEM, KBL, HBM and JLC conceived the idea and obtained funding for the study. All authors designed the trial protocol and drafted the manuscript. All authors have read and approved the final manuscript.

## Supplementary Material

Additional file 1 **The eccentric calf muscle exercise instructional DVD: part 1 of 4**. The video shows the eccentric calf muscle exercise instructional DVD provided to participants: part 1 of 4. Click here for file

Additional file 2 **The eccentric calf muscle exercise instructional DVD: part 2 of 4**. The video shows the eccentric calf muscle exercise instructional DVD provided to participants: part 2 of 4. Click here for file

Additional file 3 **The eccentric calf muscle exercise instructional DVD: part 3 of 4**. The video shows the eccentric calf muscle exercise instructional DVD provided to participants: part 3 of 4. Click here for file

Additional file 4 **The eccentric calf muscle exercise instructional DVD: part 4 of 4**. The video shows the eccentric calf muscle exercise instructional DVD provided to participants: part 4 of 4. Click here for file
